# Comparative, Prospective, Case–Control Study of Open versus Laparoscopic Pyeloplasty in Children with Ureteropelvic Junction Obstruction: Long-term Results

**DOI:** 10.3389/fped.2017.00010

**Published:** 2017-02-01

**Authors:** Lisandro A. Piaggio, Juan P. Corbetta, Santiago Weller, Ricardo Augusto Dingevan, Víctor Duran, Javier Ruiz, Juan C. Lopez

**Affiliations:** ^1^Division of Pediatric Urology, Hospital IGA Dr. José Penna, Hospital Italiano Regional del Sur, Hospital Privado Dr. Raúl Matera, Bahía Blanca, Argentina; ^2^Division of Pediatric Urology, Hospital Nacional de Pediatría SAMIC Dr. JP Garrahan, Buenos Aires, Argentina

**Keywords:** comparative, prospective, laparoscopic pyeloplasty, open pyeloplasty, randomized, children, long-term results, ureteropelvic obstruction

## Abstract

**Introduction:**

We compare open pyeloplasty (OP) versus laparoscopic pyeloplasty (LP) in children in a multicenter, prospective, case–control study.

**Materials and methods:**

From May 2007 to March 2009, a program was established at Hospital Garrahan, the reference center, to perform LP with a mentoring surgeon that would attend the institution once a month. Every new case of ureteropelvic junction obstruction (UPJO) diagnosed in the reference institution was offered to participate in the study. If the patient was enrolled, it was scheduled for LP. The following patient diagnosed with UPJO was operated on with open technique and served as a case–control. In three other facilities, patients were only offered LP and had a matched control open case at the reference institution. The first end point of the study was patient recovery: analgesia requirement and length of hospitalization (LOH). The second end point of the study was resolution of UPJO in long-term follow-up for the two techniques. Demographic data, surgical time, perioperative complications, analgesia requirement, analgesia score during hospitalization, LOH, and outcome were recorded. Both groups received the same postoperative indications for pain control. Parents were asked to assess pain in their children every 4 h postoperatively and to complete a pain scale chart to which the nurses were blinded.

**Results:**

Fifteen OP and 15 LP were compared. Groups were similar with regard to sex, age, weight, and laterality. Mean surgical time was longer in LP than in OP group (mean 188 versus 65 min) (*p* < 0.01). Hospitalization was shorter for LP group with a mean of 1.9 versus 2.5 days for OP group (*p* < 0.05). Postoperative analgesia requirement was significantly higher in the OP group with a mean use of morphine of 1.7 versus 0.06 mg/kg in the LP group (*p* < 0.05). Pain scores were similar in both the groups. At a mean follow-up of 58 months there were no failures.

**Conclusion:**

In this prospective comparative cohort, LP was a longer procedure than OP. Both procedures had the same efficacy and complication rates, but patients undergoing LP needed fewer narcotics for pain control and had a shorter hospitalization.

## Introduction

Open pyeloplasty (OP) remains as the gold standard to repair ureteropelvic junction obstruction (UPJO) in children (1). Laparoscopic pyeloplasty (LP) is considered by many the new gold standard for adult pyeloplasty and has gained slow acceptance in pediatrics since its first report in 1995 (2). LP has been reproduced in all continents with a success rate comparative to open surgery using either the transperitoneal or retroperitoneal approach (3–7) regardless of patient age or size (8–10). Other techniques of minimally invasive surgery (MIS) like robotic-assisted LP, single site LP (SSLP), or laparoscopic-assisted pyeloplasty have demonstrated satisfactory results as well (11–13).

Most of the comparative studies between OP and LP are retrospective and often with cohorts of patients in a different timeline, making its comparison troublesome. Well-designed prospective studies and randomized controlled trials comparing OP and LP are scant (14, 15) with contradictory results with regards to patient recovery. The aim of the present study was to compare LP versus OP for primary repair of UPJO in children in a multicenter, prospective, case–control study. We hypothesize that using the same postoperative setup for the two techniques and a standardized protocol for administration of pain medication and patient discharge, patients undergoing LP would have a faster recovery.

## Materials and Methods

### Patient Enrollment

Hospital Nacional de Pediatría SAMIC Dr. J. P. Garrahan is a high complexity National Pediatric Hospital receiving patients from all over Argentina. In this reference institution, operation for UPJO is usually performed with an open procedure. From May 2007 to March 2009, a MIS program was established to perform LP with a mentoring surgeon (Lisandro A. Piaggio) that would attend the institution once a month. Every new case of UPJO diagnosed in the reference institution was offered to participate in the study. If the patient was enrolled, it was scheduled for LP. The following patient diagnosed with UPJO was operated on with the routine open technique and served as a case–control if agreement for participation in the study was obtained. All parents provided written informed consent. In three other facilities in the city of Bahía Blanca, Buenos Aires, Argentina (Hospital Interzonal General de Agudos Dr. José Penna, Hospital Italiano Regional del Sur, and Hospital Privado Dr. Raúl Matera) patients were only offered LP and operated on by the mentoring surgeon. Permission from the Ethics Committee from all participating institutions was obtained. The OP case–control patients were enrolled in the reference institution under the same protocol.

### Inclusion Criteria and Outcomes

Inclusion criteria were diagnosis of UPJO and acceptance to participate in the study. We excluded patients with solitary kidney, associated kidney stones, or comorbidities.

Ureteropelvic junction obstruction was defined as symptomatic renal colic or urinary tract infection (UTI) associated with severe upper urinary tract dilatation (SFU grade III or IV), non-improvement or worsening of prenatally diagnosed hydronephrosis after at least 6 months of follow-up in association with a differential kidney function of more than 10% on DMSA renal scan, and/or obstructive pattern on DTPA or MAG3 diuretic renogram (defined as t1/2 greater than 20 min after administration of furosemide). Success rate was defined as resolution of symptoms and marked reduction of hydronephrosis on ultrasonography (SFU grade I or II). For equivocal cases, a postoperative diuretic renogram was obtained and considered normal if the t1/2 was less than 20 min after administration of furosemide. Follow-up with clinical assessment and renal and bladder ultrasound were scheduled at 1 month after stent removal and then at 3, 6, and 12 months after surgery and then yearly.

We recorded form of presentation, demographic data, analgesia requirement, and analgesia score during hospitalization, length of hospitalization (LOH), surgical time, perioperative complications, and outcome.

### Surgical Technique and Postoperative Management

Laparoscopic pyeloplasty was performed transperitoneally with three ports as previously described (16). Kidney was accessed retroperitoneally through a flank incision in the OP group. In all cases, a dismembered pyeloplasty was performed with a 5-0 or 6-0 absorbable monofilament running suture and a double J ureteral stent; a Foley catheter and perinephric drain were placed during the procedure. If a double J ureteral stent could not be advanced, another form of renal drainage was left or a perinephric drain was not placed, and the patient was excluded from the study.

The ureteral stent was placed by cystoscopy at the beginning of the procedure in all cases of LP. For OP patients, the stent was advanced in an antegrade fashion during the procedure or by cystoscopy at the beginning of the case according to the surgeon’s preference. Surgical time was recorded separately for cystoscopy, pyeloplasty, and total operating room time (including anesthesia time for induction and recovery).

No epidural blocking or regional anesthesia was used. Local bupivacaine at 0.25% (maximum 3 mg/kg per dose) was used in all patients before incision, at the wound site for OP and trocar placement site for LP.

For postoperative pain management, the attending pediatrician prescribed 10 mg/kg of dipyrone intravenously every 6 h for all patients. For breakthrough pain, morphine or nalbuphine at 0.1 mg/kg every 4 h was indicated. Once patients started fluid intake, oral ibuprofen at 10 mg/kg every 6 h and oral codeine or morphine administered for breakthrough pain every 4 h were indicated. Doses of opioids other than morphine were converted to its equivalent in morphine and expressed in milligrams per kilogram. The need and administration of opioids was determined by the nursing staff using the FLACC Scale for children 2 months to 3 years, the Wong-Baker FACES Pain Rating Scale for ages 3–7 years, and a Numeric Scale of 0–10 for ages ≥7. Pain was assessed routinely every 2–4 h. A narcotic was administered for intermediate and high pain scores (6 and higher). The nursing staff was assessed by the pediatric unit or the pain service. Participating surgeons had no incumbency on pain medication orders or administration to avoid bias.

**Figure 1:**
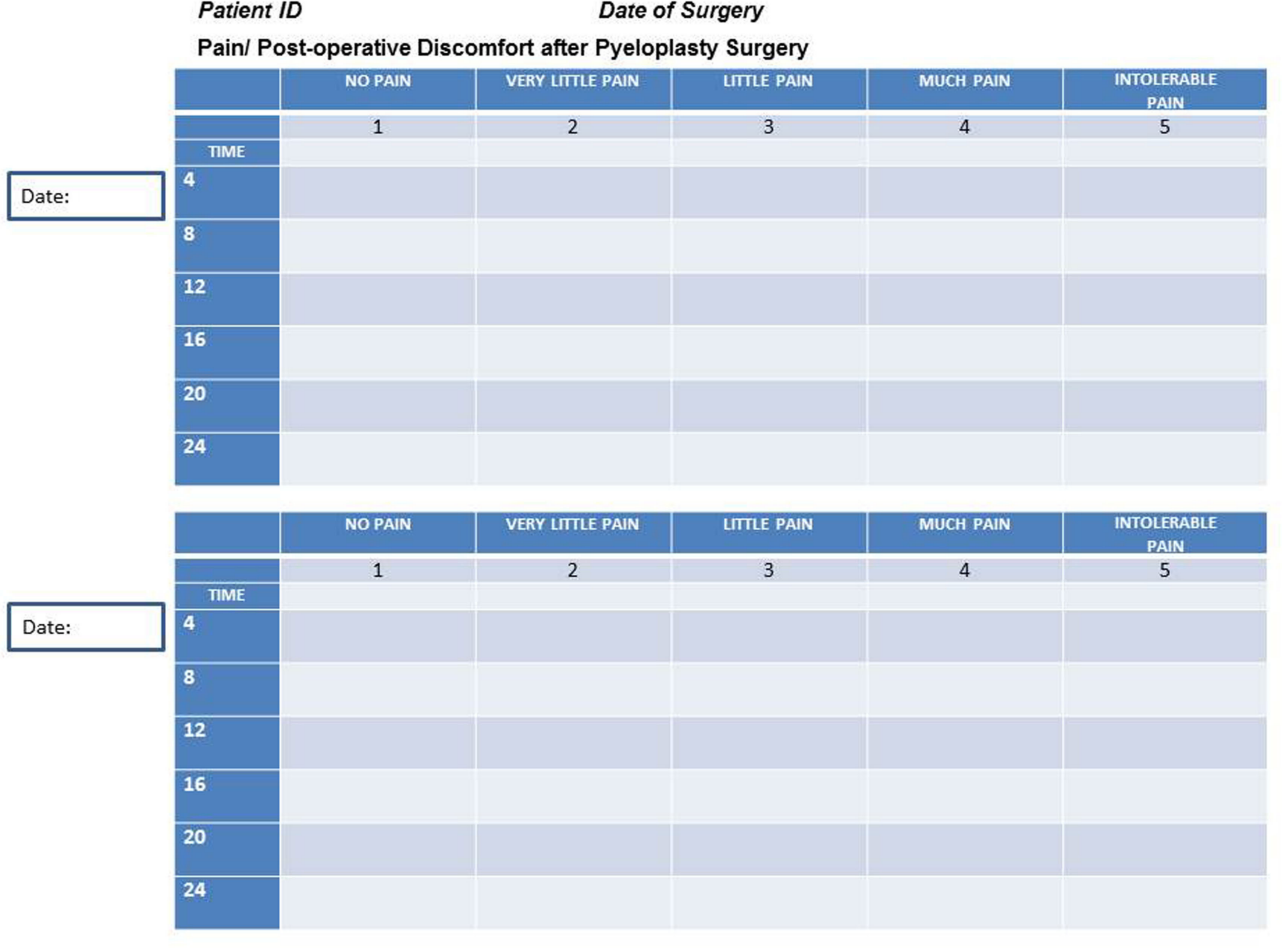
**Parent’s chart for postoperative pain assessment**.

Parents were asked to assess pain in their children every 4 h postoperatively and to complete a pain scale chart in a scale of 1 to 5 (Figure 1). The nurses were blinded to the form that parents completed. This simplified chart was retrieved in order to asses if pain management was equal in both groups regardless of nurse assessment and administration of pain medication.

Patients were discharged home if they met all of the following criteria: started enteral feeding and tolerated a full diet, tolerated oral medications with no need for narcotics for breakthrough pain, voided normally after Foley catheter was removed, and the drain was removed after minimal or if no output was recorded. Typically, the Foley catheter was removed on the first or second postoperative day after the recovery of bowel function. The drain was removed just before the patient discharge if normal voiding was assessed with no increases in drain output.

### End Points of the Study

The first end point of the study was aimed at patient recovery: analgesia requirement with a comparable pain status and LOH. We used the same “postoperative set-up” (same type of renal drainage, perinephric drain, and Foley catheter) for both the procedures and the same protocol for patient discharge. The second end point of the study was resolution of UPJO in long-term follow-up for the two techniques. Secondary end points were comparison of operating time, complications, need for secondary procedures between the two groups, and the feasibility of establishing an MIS program in a high-volume pediatric teaching facility in Argentina with no previous experience with LP.

### Statistical Analysis

Statistical analysis was performed using Microsoft Office Excel 2010^®^ data analysis. For continuous non-parametric variables, Student’s *t*-test with equal variance was used. For ordinal data, Fisher’s exact test was used. Data were expressed in mean and range for continuous variables. A *p* value of <0.05 was considered statistically significant.

## Results

Thirty-five patients accepted to be enrolled in the study. During the study period, a total of 45 patients with UPJO were operated on by the authors. Five patients were excluded: one patient in which antegrade stenting was not possible in the OP group was left without ureteral stent and excluded from the study. The other four patients were excluded from the LP group: associated kidney stones (two); solitary kidney and conversion to open surgery, one each. Of the 30 patients included, there were 15 in each group for OP and LP.

Demographic data are shown in Table S2 in Supplementary Material. Male to female ratio was 2/1 and 4/1 for LP and OP, respectively. Mean age (range) was 88 months (9–215) for the LP group and 75 months (6–204) for the OP group. Mean weight (range) was 24.9 kg (8.5–65) and 22.3 kg (10–44) for the LP and OP groups, respectively. Groups were similar with regard to sex, age, weight, laterality, and form of presentation. Antenatal hydronephrosis was the form of presentation in six and three patients in the LP and OP groups, respectively (*p* = 0.32). All patients remained with grade III or IV hydronephrosis after 6 months of follow up. Four of these patients eventually developed UTI at a mean age of 8 months (range 3–15 months). In the remaining five patients, indication for surgery were differential kidney function of more than 10% on DMSA renal scan (two) and obstructive pattern on DTPA or MAG3 diuretic renogram (three). These studies were performed at a mean age of 10 months (range 6–14 months). Patients with no history of antenatal hydronephrosis often presented with more than one symptom. Most common symptoms were recurrent flank pain and UTI, which were equally distributed in both groups (7/8 and 5/8 for LP/OP, respectively). Vomiting (2/0) and hematuria (0/1) were less common (Table S1 in Supplementary Material). Only one patient in the LP group presented with typical Dietl’s crisis.

Mean surgical time (range) for LP was 163 min (228–100) versus 80 min (65–150) for OP (*p* < 0.01). All patients in LP and 7 patients in OP underwent cystoscopy for stent placement with a mean of 29 and 33 min, respectively, including patient repositioning. Total duration of the procedure and total operative time were significantly longer for the LP group (Table S3 in Supplementary Material). There was a trend for a decrease in operative time for LP patients along the study (Figure 1). Length of hospital stay, Foley catheter, perinephric drain, and double J stent duration are summarized in Table S4 in Supplementary Material. Hospitalization was shorter for LP (*p* < 0.05). Postoperative analgesia requirement was significantly higher in the OP group for intravenous, oral, and total narcotic intake (oral plus intravenous opioid) with a total mean (range) use of morphine of 0.17 mg/kg (0.1–0.2) compared to 0.07 mg/kg (0–0.2) in the LP group (*p* < 0.01). Pain scores were similar in both the groups (Table S5 in Supplementary Material).

There were four complications in the LP group: febrile UTI (two), double J stent disruption and meatal stenosis (one each); and three in the OP group: febrile UTI, flank paint due to stent displacement, and persistent gross hematuria (one each). There was no need for transfusions in any group. Two patients in the LP group and one in the OP group needed additional procedures (*p* > 0.05): meatoplasty and ureteroscopy for stent removal (LP) and stent repositioning (OP).

At a mean follow-up time (range) of 65 (13–96) and 61 months (22–90) for LP and OP, respectively, there were no failures. All symptomatic patients are currently symptom free. Hydronephrosis decreased from grade III/IV to I/II in all except one case. One patient in the LP group who presented with flank pain persisted with grade III hydronephrosis at 6 months follow-up. The symptoms did not reoccur. A MAG 3 diuretic renogram was obtained, which showed normal washout. Postoperative DTPA diuretic renogram with normal washout were obtained in two other patients for comparison on the pre- and postoperative split renal function, but not to judged success of the pyeloplasty (highly improved postoperative ultrasound). Twelve other patients got a follow-up DMSA scan between 6 and 15 months postoperatively for comparison with the preoperative study. These findings will be the focus of a future report.

## Discussion

In the past decade, an increasing trend to minimally invasive pyeloplasties from 0.34 to 11.7% has been reported (17, 18). Despite this fact, OP remains the most common operation in children with UPJO, and MIS appears to be offered only in centers were surgeons have mastered great expertise in laparoscopic surgery, commonly in high-volume pediatric hospitals and teaching hospitals (17).

Since its first report in pediatric patients more than 20 years ago (2), LP has been reproduced all over the world, but there is still controversy whether it is a better or “less invasive” procedure than the open counterpart. Many of the pediatric patients that nowadays will be scheduled an operation for UPJO had antenatal diagnosis of hydronephrosis. Even with a conservative approach, once the decision is made for operation, the patients are small enough to be offered an open procedure with a “tiny” incision. In 2011, Ruiz et al. reported on 45 patients with a short hospital stay, no failures and no need for postoperative narcotics for pain control (19). Despite its excellent results, the study lacked a control group and did not report on a protocol for analgesia administration. It is very likely that pain is underestimated in small patients, especially if a protocol for pain assessment is not used. In our experience, all our patients with OP required opioid medication with a mean of 0.17 mg/kg of morphine equivalent for pain control despite the patient age or incision size. An analysis of a subset of patients undergoing OP under 24 months of age revealed a trend for decreased narcotics used compared to the whole group (mean of 0.12 mg/kg morphine equivalent), but still all patients needed narcotics for pain control. The comparison of patients under 2 years of age between LP (four) and OP (seven) showed a trend for a decreased use of narcotics in the LP group (0.05 mg/kg); however, there are very few patients to draw a conclusion.

The argument in favor of performing an OP in small children claims that this operation can be achieved through a small incision, 2–3.5 cm, with excellent results (19, 20). It can be argued that 3.5 cm is a big incision in a baby in proportion to its body size. Scars as small as 1.5 cm grow with patient development and can be easily identified in an adult patient. Depending on body localization, this can be upsetting, especially for girls. In our study, we demonstrated that even “small incisions” for OP operations cause more pain than LP. It is interesting to notice that authors who have reported on “minimally invasive open pyeloplasty” (20) have changed to LP a few years later (21).

Since the alleged advantages of laparoscopic surgery, namely magnification, excellent visualization, minimal blood loss, less surgical scarring, and improved cosmetics, are mostly subjective parameters difficult to correlate with a real benefit for the patient, we conducted the present study focusing the results on patient recovery. We hypothesis that using the same postoperative setup for the two techniques and a standardized protocol for administration of pain medication and patient discharge, patients undergoing LP would have a faster recovery. We proved that with the same success rate (100% in this cohort), postoperative complications, and need for secondary procedures, LP had a significantly shorter LOH and decreased use of narcotics for pain control compared to OP. Our findings are congruent with the majority of the current literature (1, 15, 22, 23). Of note, participating surgeons had no incumbency on pain medication orders to avoid bias. Postoperative indications were placed by the attending pediatrician and pain medication administered at stuff nurse discretion using validated pediatric pain scores. Furthermore, this study is unique on parents’ “double check” of pain status. The pain scale chart the parents were asked to complete in a scale of 1 to 5 (Figure 1), although it has not been validated yet, offers reassurance of the study results. Neither surgeons nor parents, nurses, or pediatricians could be blind to the type of surgery performed in the child; however, both pediatricians and nurses who placed orders and administered pain medications were blinded to the parent’s chart. There was no statistical significance (*p* = 0.28) in the pain scale charts with a mean score of less than 2, “very little pain,” in both groups, which means that pain control was certainly achieved. Both groups had comparable distribution with a mean score for pain slightly higher in the OP group, which is consistent with the group that needed more narcotics for pain control.

Mean LOH was significantly shorter for LP compared to OP, with a mean time of 1.8 days (range: 1–4) and 2.5 days (range: 2–4), respectively (*p* = 0.03). Patients were discharged from hospital with the approval of pediatricians and surgeons. There may be a potential bias in sending patients home earlier with the “less invasive procedure.” Since the same criteria were used for patient discharge including no need for narcotics for pain control, this strengthened the point that LP convalescence is shorter. Penn et al. reported no difference in LOH and analgesic usage between OP and LP in children in a prospective randomized trial (14). In their study, criteria for patient discharge and for the use of narcotics were not specified. This may explain the difference encountered in the studies. If the patients undergoing OP in our study have been sent home with oral narcotics, the LOH may have been the same than patients undergoing LP, and the difference in the use of oral narcotics unnoticed.

The transition from OP to a MIS technique is a challenging process, but it is very likely that once the surgical team feels comfortable with a new procedure, the OP will be abandoned. Most of the reports on LP are retrospective series, innovations, or variations in the technique (for instance, SSLP) (12, 13). The majority of the comparative studies between OP and LP are retrospective in nature and often with cohorts of patients in a different timeline, making its comparison troublesome especially if the team’s learning curve for LP is included in the comparison with a well-established open procedure (12, 16, 22–24).

We have reported a retrospective comparative study in 2007 in which “patient set-up” and patient age was different in both groups (16). It was sensitive that a prospective comparative study controlling the same intra and postoperative variables was in need. The first author’s preference was to perform LP for UPJO in all age groups. The difficulty in getting an OP control group was overcome with the addition of another institution with no previous experience in LP. While a MIS program was established to perform LP, routine OP procedures were continued to be offered and served as a control group with the “same set-up.” The MIS program was abandoned at the end of the study and reestablished a few years later. At present, the Urology department of Hospital Garrahan routinely offers LP for children older than 3 years of age. Facts that influenced this change in practice in addition to the present study were current fellows have a background in advance laparoscopic surgery, staff members trained in hands-on courses of laparoscopy available in the reference institution, current trends in MIS, and more confidence in performing the procedure and its results.

A limitation of our study is the small number of patients in each group. Nevertheless, statistical difference was encountered for the primary end point of the study. Another weakness of the study is that randomization was not possible, and the cases were not consecutive, since the reference institution could only offer LP on a monthly basis with a mentoring surgeon. During the study period, 5 patients were excluded from the study and 10 patients diagnosed with UPJO were not offered enrollment and operated on with open surgery. Every LP case operated on in any of the participating institutions had a consecutive OP control at Hospital Garrahan that served as a matched control in a prospective manner. All the OP and about one-third of the LP were performed in this institution. The rest of the LP patients were operated on in institutions were only LP was offered. The groups had no statistical difference as regards sex, age, weight, and laterality, making them similar for comparison.

**Figure 2:**
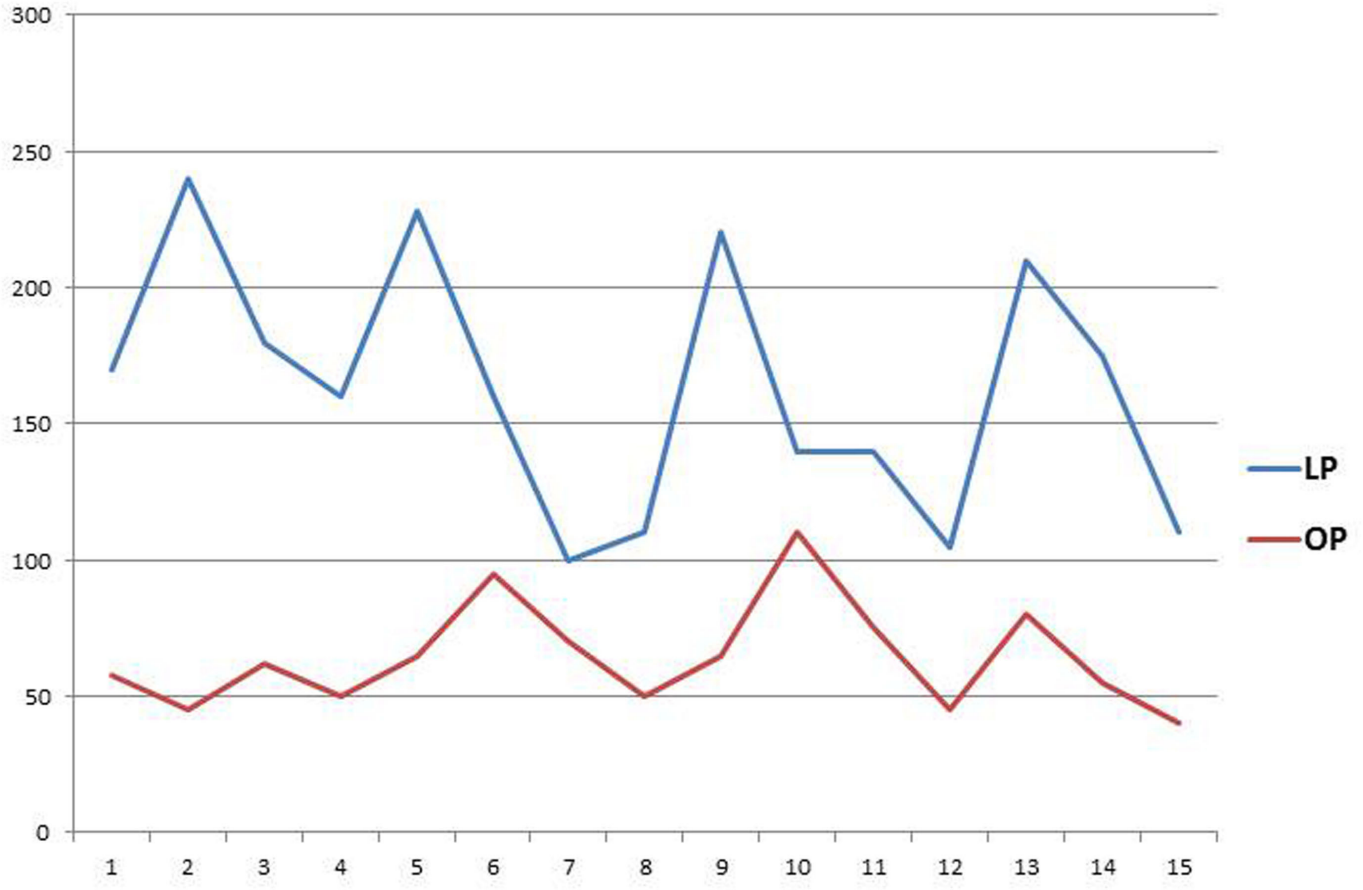
**Operative time**. *References*: *Y* axis, minutes; *X* axis, case number; LP, laparoscopic pyeloplasty; OP, open pyeloplasty.

Operating time for LP was longer compared to OP, regardless of cystoscopy added time. Our findings are in agreement with most of the reports in the literature comparing MIS techniques to OP (1, 14, 15, 25, 26). Even though the surgeon performing the laparoscopic cases had previous experience with this operation, there was a trend to decreased operative time among cases (Figure 2).

Different approaches of MIS for UPJO, such as robotic-assisted pyeloplasty (ROP), SSLP, or laparoscopic-assisted pyeloplasty have been reported with similar results to those of OP (11, 12, 25, 26). Laparoscopic-assisted pyeloplasty makes sense for those who have not mastered laparoscopic intracorporeal suturing, facilitating ureteropelvic junction dissection under laparoscopic magnification and a great operating field and making a hand-sewn anastomosis through a small incision (13, 27). ROP compares favorably to OP, but it is equivalent to LP as to patient recovery and carries larger incision sites and increased costs. It may be suitable for adult-size patients; however, while instruments are not miniaturized and costs decreased, it does not prove appropriate for small size patients (17, 26, 28).

Laparoscopic pyeloplasty has been described through a transperitoneal and retroperitoneal approach (3–6) regardless of patient age or size (8–10). We believe the transperitoneal approach is suitable for all patients with an excellent operative field and a short convalescence. Although subjective, the cosmetic appearance of the anterior abdominal incisions for transperitoneal LP (often unnoticed) compares favorably to the “more visible” lateral scars. In a comparative study, Canon et al. found that LP has decreased operative time, less conversions and complications that retroperitoneoscopic pyeloplasty, and they recommend the first approach because of the larger working space, ease of antegrade stenting, and improvement in cosmetic outcome (29).

The second end point of this study was resolution of UPJO in long-term follow-up for the two techniques. There have been reports of long-term recurrences of UPJO (30). Since the follow-up period of this cohort of patients is longer than 5 years, we believe it is fair to assume there are no recurrences on the long term.

## Conclusion

Dismembered pyeloplasty is a highly efficient procedure to treat UPJO in children either with an open or laparoscopic approach, with an overall complication rate of 20% in our series. Additional procedures other than stent removal in this study were performed in three patients (10%). These figures may help in parents’ counseling before surgery.

In this prospective comparative cohort, LP had comparable complications rate and need for secondary procedure than OP with the same success in a long-term follow-up. Patients undergoing LP had a faster recovery: decreased need for narcotics for pain control and shorter hospitalization.

In the long term, an MIS program for LP was established in an important tertiary facility in Argentina.

## Ethics Statement

Comité de Ética en Investigación Sobre Seres Humanos, Hospital Interzonal Dr. José Penna. Comité de Docencia e Investigación, Hospital Privado Dr. Raúl Matera. Comité de Docencia e Investigación, Hospital Italiano Regional del Sur. Comité de Ética en la Investigación, Hospital de Pediatría SAMIC, Prof. Dr. Juan P. Garrahan. All parents provided written informed consent for the study and for the surgeries performed.

## Author Contributions

LP: study idea, study design, data collection, manuscript preparation, manuscript revision, patient’s interventions, and follow-up. JC: study design, data collection, manuscript preparation, patient’s interventions, and follow-up. SW: patient’s intervention and follow-up. JR: data collection, patient’s interventions, and follow-up. RD: patient’s intervention and follow up. VD and JL: study design and patient’s interventions.

## Conflict of Interest Statement

The authors declare that the research was conducted in the absence of any commercial or financial relationships that could be construed as a potential conflict of interest.
