# Supplementary material for: Comparative, Prospective, Case–Control Study of Open versus Laparoscopic Pyeloplasty in Children with Ureteropelvic Junction Obstruction: Long-term Results
Source: Front Pediatr. 2017 Feb 1;5:10. doi: 10.3389/fped.2017.00010 (PMC5285361; doi:10.3389/fped.2017.00010)
Supplement: Supplementary file 1 [file Table_1.PDF]

	LP<br>n = 15	OP<br>n = 15	P value
Antenatal hydronephrosis	6	3	0.31
Recurrent flank pain	7	8	0.54
UTI	5	8	0.36
Vomiting	2	0	0.27
Hematuria	0	1	0.51

Table 1: Form of Presentation
