# Supplementary material for: Comparative, Prospective, Case–Control Study of Open versus Laparoscopic Pyeloplasty in Children with Ureteropelvic Junction Obstruction: Long-term Results
Source: Front Pediatr. 2017 Feb 1;5:10. doi: 10.3389/fped.2017.00010 (PMC5285361; doi:10.3389/fped.2017.00010)
Supplement: Supplementary file 2 [file Table_2.PDF]

	LP<br>n = 15	OP<br>n = 15	P value
Females/ Males	5 / 10	3/ 12	NS
Left side/ right side	12 / 3	10 / 5	NS
Mean age in months (range)	88 (9 –215)	75 (6-204)	NS
Mean weight in Kg (range)	24.9 (8.5-65)	22.3 (10 -44)	NS

NS: not significant

Table 2: Patients' Characteristics
