# Supplementary material for: Comparative, Prospective, Case–Control Study of Open versus Laparoscopic Pyeloplasty in Children with Ureteropelvic Junction Obstruction: Long-term Results
Source: Front Pediatr. 2017 Feb 1;5:10. doi: 10.3389/fped.2017.00010 (PMC5285361; doi:10.3389/fped.2017.00010)
Supplement: Supplementary file 3 [file Table_3.PDF]

Mean duration of procedure in minutes (range)	LP<br>n = 15	OP<br>n = 15	P value
Pyeloplasty	163(110-228)	64 (40-110)	< 0.01
Cystoscopy Stent and positioning	29 (15-52)	33 (15-45)<br>(n = 7)	NS
Total operative room time	250 (140-360)	106 (65-155)	< 0.01

Table 3: Surgical Time
