# Supplementary material for: Comparative, Prospective, Case–Control Study of Open versus Laparoscopic Pyeloplasty in Children with Ureteropelvic Junction Obstruction: Long-term Results
Source: Front Pediatr. 2017 Feb 1;5:10. doi: 10.3389/fped.2017.00010 (PMC5285361; doi:10.3389/fped.2017.00010)
Supplement: Supplementary file 4 [file Table_4.PDF]

	LP<br>n = 15	OP<br>n = 15	P value
Mean Foley duration in days (range)	1.4 (1-3)	1.5 (1-3)	NS
Mean duration of drain	1.8 (1-4)	2.5 (2-4)	0.03
Mean hospitalization in days (range)	1.8 (1-4)	2.5 (2-4)	0.03
Postoperative complications	3	3	NS
Need for secondary procedures	2 ¤	1*	NS

¤ Meatoplasty, ureteroscopy for stent removal
* Double J Stent repositioning

Table 4: Hospitalization course, readmissions and complications
