# Supplementary material for: Comparative, Prospective, Case–Control Study of Open versus Laparoscopic Pyeloplasty in Children with Ureteropelvic Junction Obstruction: Long-term Results
Source: Front Pediatr. 2017 Feb 1;5:10. doi: 10.3389/fped.2017.00010 (PMC5285361; doi:10.3389/fped.2017.00010)
Supplement: Supplementary file 5 [file Table_5.PDF]

	LP<br>n = 15	OP<br>n = 15	P value
Mean IV morphine mg/kg (range)	0.06 (0-0.2)	0.12 (0.05-0.15)	<0.01
Mean oral narcotics mg/kg (range)	0.01 (0-0.05)	0.05 (0-0.1)	<0.01
Mean narcotic requirement: oral + IV (range)	0.07 (0-0.2)	0.17 (0.1-0.2)	<0.01
Number of patients without narcotic requirement	7	0	<0.01
Average Pain Score from parent's chart (range)	1.7 (1-3.2)	1.98 (1-3.2)	0.28

Table 5: Analgesia Requirement
